# Qualitative study of knowledge and attitudes to biobanking among lay persons in Nigeria

**DOI:** 10.1186/1472-6939-13-27

**Published:** 2012-10-16

**Authors:** Michael A Igbe, Clement A Adebamowo

**Affiliations:** 1 Department of Public Health, Federal Ministry of Health, 9th floor, Room 909, Federal Secretariat, Phase 3, Abuja, Nigeria; 2 Department of Surgery, Faculty of Clinical Sciences,West African Bioethics Training Program, University of Ibadan, Ibadan, Nigeria; 3 Department of Epidemiology and Public Health, Institute of Human Virology and Greenebaum Cancer Center, University of Maryland, Baltimore, USA; 4 Institute of Human Virology, CBD, FCT, 252 Herbert Macaulay Way, Abuja, Nigeria

**Keywords:** Biobanking, Non-communicable diseases, Public perspectives, Nigeria

## Abstract

**Background:**

Interest in biobanking for collection of specimens for non-communicable diseases research has grown in recent times. This paper explores the perspectives of Nigerians on donation of specimen for the biobanking research.

**Methods:**

We conducted 16 Focus Group Discussions (FGD) with individuals from different ethnic, age and socio-economic groups in Kano (North), Enugu (Southeast), Oyo States (Southwest) and Abuja, the Federal Capital Territory (Central) of Nigeria. We used topic guides and prompt statements to explore the knowledge and understanding of interviewees to general issues about biobanking of biospecimens, their use and specifically about role of biobanking in non-communicable diseases research.

**Results:**

A total of 123 individuals participated in 16 focus group discussions in 2011. Our participants had limited knowledge of the concept of biobanking but accepted it once they were educated about it and saw it as a worthwhile venture. Half of our study participants supported use of broad consent, a quarter supported restricted consent while the remaining quarter were in favour of tiered consent. Most discussants support shipment of their samples to other countries for further research, but they prefer those collaborations to be done only with competent, ethical researchers and they would like to receive feedback about such projects. The majority preferred health care as a benefit from participation, particularly for any unexpected condition that may be discovered during the course of the research instead of financial compensation. Participants emphasized the need to ensure that donated samples were not used for research that contradicts their religious beliefs.

**Conclusions:**

Our study demonstrates that our participants accepted biobanking once they understand it but there were different attitudes to elements of biobanking such as type of consent. Our study highlights the need to carefully document population attitudes to elements of modern scientific research and the consenting process.

## Background

Biobanking is now a major element of modern biomedical research and was recognized by Time® magazine as one of the ideas changing the world right now in 2009. Nevertheless, a number of concerns have been raised about related issues such as commercial genetic research, commodification of human tissue, benefit-sharing, risk to society, eugenics, fairness, autonomy, dignity and trust.

All these problems seem, however, to have been resolved through a particular “solution”-informed consent [1]. The principle of informed consent is considered a pillar of the practice of research ethics [2]. Informed consent defines the “moral contract” between researchers and the study participants, and sets the framework for the allowable use of biospecimens and data [3]. The concept of informed consent evolved gradually over the years, typically in response to major instances of research ethics violations. For example the Nuremberg Code followed the inhuman experimentations carried out by Nazi doctors during the Second World War. More recently the foundations of informed consent are being laid by philosophical reflection and empirical research outside of the context of research misconduct.

While data and sample sharing is essential to accelerating scientific discovery, it presents a number of challenges, one of which is balancing the interests of science, the scientist, and the donor. For example, when researchers want to use samples for new purposes, the onerous task of seeking re-consent from donors may slow the process of scientific discovery, yet it is critically important to maintain public trust in the scientific process by ensuring that research is done ethically [4].

A characteristic of biobank is the potential that the resources donated to the biobank would be distributed to researchers in different parts of the world, far from where the samples were collected [5]. As research has become increasingly globalised, the ethical issues arising from international collaborative research where samples are collected in developing countries and exported to developed countries for analysis are particularly complex. Such international research raises concerns about exploitation, the validity of informed consent from populations with low levels of education and high prevalence of poverty, appropriate benefit-sharing between sponsors and participants, and guidelines for regulating types of future research [6-9].

Secondary uses for stored human samples are nearly always possible even though they are usually not foreseeable at the time of sampling [10-16]. The main ethical issues relate to the level of completeness of the information given, the necessity or not of obtaining a new individual consent for each use, and who decides on these issues [2]. The collection, storage and use of biological samples in future research raise unique ethical and policy issue and there has been no consensus on these issues among different national and international guidelines [17-19]. Chief among these ethical issues include questions regarding confidentiality, ownership and the commercialization of stored biological samples.

In the context of biobanks, protection of the person is practically synonymous with controlling access to the data and use of such data. This ensures that individuals or groups are not discriminated against and that medical and personal information are not disclosed to third parties (such as other family or community members, colleagues, employer or insurance companies). Protection is a central issue in ethical analyses related to biobanks [11-13,15,20-23] and it is believed that this is best achieved through collection of anonymous data [11,12,24]. The expansion of biobanks to developing countries raises urgent need for exploration of the ethical issues that arise in these contexts.

## Methods

We conducted Focus Group Discussions (FGD) with individuals from different ethnic, age and socio-economic groups in Kano (North), Enugu (Southeast), Oyo States (Southwest) and Abuja, the Federal Capital Territory (Central) of Nigeria in January 2011. We used topic guides and prompt statements to explore the knowledge and understanding of interviewees to general issues about biobanking of biospecimens, their use and specifically about role of biobanking in non-communicable diseases research.

Kano State was chosen to cover the predominantly Muslim North where Hausa/Fulani are the major ethnic group, Oyo and Enugu covered the predominantly Christian Southern part with Yoruba and Igbo as the major ethnic groups respectively while the Federal Capital Territory (FCT) is more cosmopolitan with a mixture of ethnic groups but the majority are Gwari. Participants were selected through community contacts that identified community leaders, spokespersons and traditional religionists in their communities. One FGD was conducted for males and a separate one for females in 2 different settings - one rural area, one urban area (See Table 1). Each FGD consisted of 8 to 10 participants and was balanced in terms of gender, age, socio-economic status and religious affiliation. The FGD were moderated by MAI with interpretation by native speaker of local language where necessary. The FGDs were audio-taped and lasted an average of one hour. Focus group members signed a consent form and provided demographic information. The study was approved by the National Health Research Ethics Committee (NHREC) of the Federal Ministry of Health of Nigeria.

**Table 1:** Matrix of focus group discussion participants’ selection among the males/female residing in Urban and Rural Settings in each of Enugu, Kano, Oyo States and FCT, Nigeria, 2012

**Level of Literacy**	**1 Urban LGA**		**1 Rural LGA**		
	**Young**	**Adult**	**Young**	**Adult**	
	**Male/Female**	**Male/female**	**Male/female**	**Male/female**	
**Literate**	**2**	**2**	**2**	**2**	
**Illiterate**	**2**	**2**	**2**	**2**	
**Total**	**4**	**4**	**4**	**4**	

## Result and discussion

There were 123 participants in the FGDs. Their ages ranged from 18 to 70 years (mean 36.2 years). There were almost the same number of females (62, 50.4%) and males (61, 49.6%). Most, 84(68.3%) were Christians while 39 (31.7%) were Muslim. Their occupations were students (19, 15.5%), housewives (20, 16.3%), business persons (24, 19.5%), farmers (18, 14.6%), civil servants (21, 17.1%) while the rest (21, 17.1%) belonged to a wide variety of occupation.

### Perception and willingness to participate in biobank research for non-communicable diseases

The majority believed that research is for solving problems. One of the male focus group participants said,

“Research is used to find out the infections that are worrying people which ordinarily cannot be seen except through research.”

In the words of another participant,

“What I understand about research is that it is aimed at getting to know the type of diseases affecting the people, so as to know how to treat the diseases.”

FGD participants welcomed the idea of biobanking for research and saw it as a worthwhile venture. Only one participant had ever heard of biobanking before this research. A few participants from the northern part of the country referenced the 1996 Trovan Clinical Trial experience in Kano and said that since biobanking as explained to them was different from drug trials, they were ready to participate in its use in non-communicable diseases research.

Majority of the discussants had deep seated feelings about the value of biobanks and research and perceived it to be a good instrument for preserving specimens for biomedical research. They viewed biobank as something that will help improve human health and bring about scientific, technological and medical sciences advancement.

Other participants recognized the prospects of drugs discovery that would benefit them or their family members and future generations. In the words of a male participant,

“After the research have been carried out, drugs could be developed that can impact positively on the society.”

Another participant said,

“It can help us know our problems and can help generations to come.”

One of the female participants opined that if someone has a problem he/she can approach the biobank to research on the problem. She said,

“Biobank can help save life. If you have biobank close to you and you have a problem, you can approach them to research on your problem.”

Some participants were concerned about the benefits of participating in biobanking. A female participant said,

“What are the benefits that we will get for participating in it”?

Participants’ understanding of and attitudes to research on non-communicable diseases research were quite varied. The majority had good understanding of non-communicable diseases, while a few did not. A participant said,

“Non-communicable diseases are not transferable but are difficult to heal.”

### Informed consent and perception of the types of consent procedures

Half of the discussants supported broad consent, a quarter supported restricted consent while the remaining quarter were in favour of tiered consent.

A supporter of broad consent said,

“Since I have faith in the research and gave my specimen, I will give broad consent.”

Another supporter of broad consent said,

“I will prefer to give broad consent so that the aims and objectives of keeping samples in the biobank can be achieved.”

Even among those who supported broad consent, there was desire for a level of control over the interaction between the researchers and research participants. One of those that supported broad consent said he will give broad consent only after the Nigerian Federal Ministry of Health approves the research.

Participants who preferred tiered consent believed that giving consent for each research would allow their views on the aspect of the research they prefer to be taken into consideration by the researchers. One of the focus group members that preferred restricted consent said,

“I prefer the restricted because it will enable me have a grip of the situation and be certain of receiving a feedback.”

Another one of those that preferred tiered consent said,

“I would prefer you come back to me for re-consent, because you may be doing something that will not favour me.”

In general, those participants who preferred tiered consent did so because of a desire to have control over the research studies that their samples are used for.

Respondents had a nuanced viewed of informed consent and the dynamics of interactions between researcher and research participants. A participant who preferred tiered consent said she would first give tiered consent and if she is pleased with the conduct and outcome of the initial research, she will go ahead to give the broad consent, otherwise she will withdraw from the study altogether. Many of the participants expressed support for this approach.

### Specimens and data sharing with commercial and non-commercial entities over time

All the focus group participants agreed to share their specimen with other researchers, provided that there are guarantees in place to prevent unethical research being conducted on their specimens, those running the biobank are ethical, trustworthy and competent. A minority of participants would like other researchers who want to use their samples to approach them for their consent.

A significant minority was worried that sharing their specimens with researchers outside Nigeria could lead to conduct of research that will further stigmatize Nigerians internationally and worsen discrimination against Nigerians. One of them said,

“I wouldn’t like you to give them our specimen as they will discover things about us and begin to discriminate against Nigerians”.

In the words of another male focus group participant,

“If you are sure that they are genuine researchers, you can give them my specimen, but they should not use it for animal research.”

Another participant said,

“My opinion is that the other researchers should be competent and from reliable institutions and countries. Once we are sure their objectives are okay, I will agree.”

Most of the focus group discussants do not mind shipment of their samples to other countries for further research, but they would want those collaborations to be done with only competent researchers and they would like to receive feedback about such projects. Only one respondent preferred that her samples be shared only with researchers in the United States because she believes that researchers there are more competent. In her words,

“I prefer that my specimen be taken to America because they can best discover causes of sicknesses.”

Another female said,

“My specimen can be taken to any country, provided the researchers are competent.”

A few discussants preferred local researchers to international for reasons of proximity. A female said,

“I prefer my specimen to be given to a local researcher. He is nearer to me and he will be able to relate with me. I can also ask him for my result.”

Another participant said,

“If my sample is taken abroad, I would like to know the outcome of research done on it.”

A few participants made reference to the issue of observance of religious norms in the study. One of the participants said,

“You can take my samples abroad for further analysis provided it will not be used for something that is against my Christian religion.”

Another male participant said,

“If my sample is used for research that contradicts my religion, it will withdraw my participation.”

### Secondary use of specimens

The majority of the participants do not mind if their specimens are stored for future unspecified research. Only a few would like to re-consent before their specimens are used for future studies. They were also discussants who chose restricted and tiered consent. They want to be sure of the kind of research that will be done on the stored samples before they give consent. One of the participants said,

“I should be informed before other studies are done with my specimen in the future.”

### Receiving news of research and return of results

We probed participants about whether they would like to be contacted if something serious was discovered about them during the course of the research, a large majority of the discussants answered in the affirmative. Their reasons included the possibility of access to beneficial new medical knowledge and that it was better to know about their health status rather than be caught unawares particularly if the opportunity exists for advance notification based on research results. They believed that researchers would give them good quality information where applicable and care if possible. A few participants envisaged the possibility of using information about their health status derived from research for a change of life style. One of such persons said,

“I will want feedback, for example if they discover HIV in me, I will get myself prepared for death or take precautions so that I will live longer than the time I would have died.”

A female participant said,

“If something serious is discovered about me I should be told and I will not feel happy if you do not give me my results and general news about the biobank research.”

They also reiterated the need for counseling before feedback is given. In the view of a male participant,

“I should be given my individual result as well as general feedback, but with prior counseling. The feedback will show the progress that the researchers are making.”

Another participant said,

“The researchers should study someone’s mood and to see if he/she can accommodate the news before telling him/her.”

A few of the focus group discussant would not like to receive their results for reasons of fear. A female participant said,

“I would not like to know my result because I will die of fear.”

Other participants were worried that individual results or community feedback may be leaked or published by the researchers that will place someone or the community in danger of discrimination and cause dignitary harm. One of the male participants said,

“Provided the individual results or community feedback will not be leaked or published by the researchers in a way that will place someone or the community in danger of discrimination for example at the time of employment.”

### Medical records access

The majority of the discussants were ready to provide access to their medical record. Only a few participants would like to be contacted again to re-consent for access to their medical records. A few participants were initially cautious about granting access and would want to know more about what the medical records will be used for before granting access. One of the females that favoured providing medical access said,

“The fact is that I have given you my consent, I should not worry about your having access to my medical records.”

### Persons that will be informed before participating in the research

The majority of the participants would like to inform their spouses and children before they participate in biobanking. The younger ones would like to tell their parents, brothers and or sisters. A male focus group participant said,

“I will inform my wife and children.”

A female participant said,

“I will tell my husband before I participate. In fact, he will not be happy if I hide it from him.”

A few of the participants stressed the value of community assent within the context of individual consent. One of the participants said,

“The community leader and the community health officer should be informed on behalf of the community. The moment they permit the research, all the community members will respectfully participate”.

Another supporter of community leaders’ assent said,

“Like my colleague just mentioned. It is the explanation of the community leader and health worker that will be well taken when you come for such a research.”

In contrast, a female participant said,

“I will not seek anybody’s opinion before I participate, because what is good is good. It is good for the world.”

### Religious and cultural belief on biobanking research

All the focus group participants had no religious or culturally based views on biobank research. In the words of one respondent,

“As a traditionalist, I have nothing against the biobank.”

Another participant said,

“There are portions in the Quran where we are told to go and obtain health.”

Another focus group discussant said,

“If one is not well he cannot worship God properly.”

### Concerns about biobanking research

The major concerns of the participants about biobanking typically revolve around confidentiality and the problem of availability of infrastructure (such as power supply) to support such research in Nigeria. A male participant said,

“I will be concerned if the information gathered from us will not be kept confidential.”

A female participant said,

“My concern about the biobank research is the erratic power supply in Nigeria”.

A few of the participants were worried about the problem of inadequate maintenance culture and the fear that the biobank might not be sustained thereby rendering their participation a nullity. One of the male participants said,

“My concern is that the researchers can build our hope and later abandon the research. They may be unable to maintain the equipment. I am advising that adequate care should be taken on the equipment.”

A female participant said,

“My worry is that if you go to most health facilities in Nigeria there are many equipment that are not maintained or put to use. I hope it will not be the same thing with the biobank. Are we developed enough in this country to have a biobank?”

A male participant was concerned that researchers might use the samples to make money. In his words,

“I hope the research is not to achieve personal interest but the interest of the public. There was a time I donated blood free, but it was sold by the hospital. I hope this will not be the same thing.”

## Conclusions

Our study shows that there is limited knowledge of biobanking and its implications in Nigeria. Nevertheless, majority of our participants believed that biobanking is beneficial and recognized the prospects for drugs discovery that could benefit them, their family members and future generations. We also found that some of our participants wanted to know the benefits that will accrue to them from participating in the research. They rarely mentioned money as expected benefit but rather health care, particularly for any unexpected condition that may be discovered during the research.

Many, but not all discussants in this study, viewed research participation as a duty, as a chance to help. This agrees with other studies in U.S., Canada, the UK, Norway, Sweden, Austria and France [25]. Other studies have highlighted how people in Europe often see participation as a sort of obligation that goes along with benefiting from universal healthcare and medical science [26,27].

When the focus group participants were asked to choose between broad, tiered, or restricted consent, half of the respondents chose broad consent, while others chose restricted and tiered consent for biobank. The main reason for choosing tiered consent was a desire to maintain control over the types of research conducted with donated samples. Many biobanks ask participants to provide broad or “blanket” consent at enrolment [28-30]. According to the World Health Organization, “blanket informed consent - is the most efficient and economical approach, avoiding costly re-contact before each new research project” [31]. This approach reduces the financial and logistical barriers to researchers, and the burden to participants. However, recent technological advancements may enable participants to exert control on the use of their donated samples in an affordable manner [32].

All our focus group participants were willing to share their samples with other researchers, provided the specimens are used for ethical research which does not conflict with their religion. The repeated reference to religion by focus group participants highlights the high level of religious observances in the study population. Biobank research can create moral harm when samples are used for research that the participants would ordinarily strongly object to. It would be important to understand potential interaction between religious beliefs and research objectives when participants are being consented to donate samples to biobanks.

A characteristic of biobank is the potential that the resources donated to the biobank would be distributed to researchers in different parts of the world, far from where the samples were collected [5]. Majority of the discussants did not mind shipment of their samples to other countries for further research, provided they are given feedback from such collaborations and the sharing is with ethical and competent researchers. A few participants were worried that sharing their samples internationally could lead to discoveries that could lead to further stigmatization and discrimination of Nigerians.

The majority of the participants do not mind if their specimens are stored for future unspecified research but a few would like to be re-consented. When establishing the collections of samples and related data, it is impossible to anticipate the studies that might emerge in future which has implications for the precise boundaries of the consent that individuals are giving for future unspecified use research [3]. Indeed, a major ethical problem for prospective biobanks is how to assure participants’ consent when it is not known what they are consenting to in terms of future research. The question of the importance and meaning of informed consent is one main reason why international guidelines on biobanks lack consensus on this matter [3]. Views about appropriate consent required to store biological samples and data for future research range from denying any use, other than that initially stated, to more flexible attitudes [33,34]. The latter take into account the traceability or not of the individual identity, the kind of further uses that are envisaged in relation to the original one, the implications of the research for the individual (so-called ‘minimal risk’ research being more easily allowed), how precisely the use was described at the time of sampling and, finally, the kind of consent that was originally granted [34].

A common feature of all recommendations and regulations on this issue is that any unplanned use requires an authorization, with or without a new consent, by an independent research ethics committee [11,15,21,35,36]. The ethics committee can make authoritative decisions alone or in consultation with another administrative authority [34]. In addition, known future uses of specimens can be included on the current consent form in addition to the right of donors to refuse consent to the storage or future use of their samples.

Most discussants expected some feedback from the biobank on matters relevant to their individual health and as well as developments in the research even if something serious was discovered about them. They also reiterated the need for counseling before feedback is given. The traditional practice of not giving research participants any information from a study that relates specifically to them is being re-evaluated in the context of genetic research [37]. Many commentators now argue that there are ethical and legal obligations to give individuals their test results under some circumstances [32,38], for instance, when results could have an impact on their health or reproductive decisions.

Due to the nature of genetic data, genetics research on biobank samples carry significant risks of discrimination or stigmatization. Some of our participants were concerned that the publication of individual results or community feedback may place someone or the community in danger of discrimination and stigmatization. This suggests that the value of individual results and potential risks of stigmatization requires careful consideration before conducting genetic research on banked samples in our environment. These risks must be recognised, both with respect to recruitment and interpretation of results, by properly informing participants and by taking action to avoid or minimize their occurrence [2].

The majority of the participants would like to inform their spouses and children. The younger ones would like to tell their parents, brothers and or sisters. While it is generally acknowledged that individuals should be respected as autonomous agents, [39] nevertheless, our participants would discuss their decision to participate in research with others because if the participation leads to negative consequences they will need to rely on these individuals.

Participants were especially concerned about confidentiality. The majority of the discussants were ready to provide access to their medical record, while recognizing that personal information is sensitive and inadvertent release can cause harm [39,40]. Participants were also concerned about commercialization of samples and the research that they are used for. The commercial aspects of biobanks is well recognised despite the principle that “biological materials should not, as such, give rise to financial gains”, as written in the Council of Europe recommendation [30,41-43]. Any potential or actual commercial use of banked samples should be discussed in detail with participants at the time of sample donation.

Another major concern of our discussants which may be specific to low resource countries is concern about the availability of necessary infrastructure and their maintenance in order to secure long term success of the biobank and donated samples. Our focus group discussants suggested that failure to take adequate care of donated samples would be unethical and want to be reassured about these logistics of biobanking before they consent to donate their samples. In conclusion, our study shows that the population of a typical low/middle income country e.g. Nigeria, are willing to participate in biobanking but have specific ethical concerns that need to be taken into account as this resource is introduced in these environments.

## Competing interests

The authors declare that they have no competing interests.

## Authors’ contributions

All authors contributed to the conceptual development of the paper and manuscript revisions. MAI drafted the manuscript. All authors read and approved the final manuscript.

## Pre-publication history

The pre-publication history for this paper can be accessed here:

http://www.biomedcentral.com/1472-6939/13/27/prepub
